# Host Differences Affecting Resistance and Susceptibility of the Second Generation of a Pekin Duck Flock to Duck Hepatitis A Virus Genotype 3

**DOI:** 10.3389/fmicb.2017.01128

**Published:** 2017-06-19

**Authors:** Xiaoyan Wang, Jiaojiao Zhang, Runze Meng, Yong Jiang, Suyun Liang, Yunsheng Zhang, Ming Xie, Zhengkui Zhou, Shuisheng Hou

**Affiliations:** Institute of Animal Sciences, Chinese Academy of Agricultural SciencesBeijing, China

**Keywords:** duck viral hepatitis, duck hepatitis A virus genotype 3, Pekin duck, resistance breeding, host response

## Abstract

Earlier work suggested the possibility to anti duck hepatitis A virus genotype 3 (DHAV-3) using the resistance breeding strategy. Here, we report the creation of the second generations of a resistant Pekin duck flock (designated Z8R2) and a highly susceptible Pekin duck flock (designated Z8S2) and the investigation of their responses to DHAV-3. Experimental infection with DHAV-3 at 7 days of age resulted in a high mortality (66.3%) in 11 susceptible Z8S2 families and an extremely low mortality rate (2.67%) in 32 Z8R2 families, indicating that Z8R2 exhibits strong resistance to DHAV-3, while Z8S2 is highly susceptible to the virus. Detection of DHAV-3 in the liver between 1 and 60 hours post inoculation (hpi) suggests that DHAV-3 can be replicated rapidly and efficiently in the liver of Z8S2, whereas the replication of the virus in the liver of Z8R2 is suppressed greatly. High levels of serum biochemical markers (e.g., ALT, AST, ALP and GGT) were detected in Z8S2 at 24 hpi, which were significantly higher than those in Z8R2. Analysis of transcripts in the liver revealed that the expression levels of several pattern recognition receptors (PRRs) (e.g., TLR4/7, RIG-1 and MDA5) and cytokines (e.g., IL-2, IL-6, IL-8, IFN-α, and IFN-γ) in Z8S2 were significantly higher than those in Z8R2 at 12 and 24 hpi. Together these findings suggest that Z8R2 and Z8S2 Pekin ducks, which were derived from the same Z8 line, exhibit disparate pathogenic outcomes following DHAV-3 infection. Therefore, it is possible to select a Pekin duck flock resistant to DHAV-3 employing the strategy described here. It is likely that the high viral load and the strong inflammatory response correlate with the high susceptibility of Z8S2 Pekin ducks to DHAV-3.

## Introduction

Duck viral hepatitis (DVH) is a highly fatal contagious disease of ducklings, characterized by clinical signs of opisthotonos and lesions of liver hemorrhages. It usually occurs in ducklings below 3 weeks of age, and may cause up to 90% mortality if not controlled (Woolcock, 2003). Therefore, the disease is of economic importance to duck-growing farms. The causative agents of DVH include duck hepatitis A virus genotypes 1 (DHAV-1), 2 (DHAV-2), and 3 (DHAV-3), members of the species *Avihepatovirus A* of the genus *Avihepatovirus* in the family *Picornaviridae* (Knowles et al., 2012),^1^ and duck hepatitis virus type 2 (DHV-2) and duck hepatitis virus type 3 (DHV-3), which are currently classified within the genus *Avastrovirus* of the family *Astroviridae* (Bosch et al., 2011).

Vaccination is an important measure to control DVH. So far, attenuated vaccine derived from serial passages in embryonated chicken eggs has been reported for DHAV-1, DHAV-3, DHV-2 and DHV-3 (Asplin, 1965; Woolcock and Fabricant, 1991; Kim et al., 2007, 2009; Knowles et al., 2012). These vaccines have been proven to be highly efficacious in controlling DVH caused by homologous virus. However, they cannot induce cross-protection against heterologous viruses. Thus, new vaccines consisting of mixture of different strains should be developed to confer broad protection (Zhang and Liu, 2016).

Progress in some poultry diseases (e.g., Marek’s disease, Infectious bursal disease, and Salmonella infection) has proven resistance breeding an effective way to control the infectious disease (Lee et al., 1981; Bumstead and Barrow, 1993; Bumstead et al., 1993). Given that DHAV-3 is most prevalent in duck industry in East and South Asia (Fu et al., 2008; Soliman et al., 2015; Doan et al., 2016; Lin et al., 2016), the resistance breeding against DHAV-3 was started in 2014 in our laboratory by using 88 families of a Pekin duck specialized line (Z8) as a tested flock (Zhang et al., 2016). As a first step toward the resistance breeding, the authors investigated the resistance and susceptibility of Pekin duck strain Z8, a lean-type line of Pekin duck, to DHAV-3 by using experimental infections. Nine families exhibited a strong resistance to DHAV-3 infection, while 14 families were highly susceptible to DHAV-3 infection. From the investigations a susceptible flock (Z8S) and a resistance flock (Z8R) were identified and their first generations (Z8S1 and Z8R1) were created. The aim of the present study was to create the second generations of the resistant Pekin duck flock, Z8R2, and of the highly susceptible Pekin duck flock, Z8S2, and to investigate the host response to DHAV-3 infection in detail.

## Materials and Methods

### Virus

The 112803 strain of DHAV-3 was originally isolated from a 1-week-old Pekin duckling showing clinical signs and pathological changes typical of DVH in 2011 in China. The virus was propagated in allantoic cavity of 9-day-old embryonating Pekin duck eggs for 48 h at 37°C. The allantoic fluids, allantoic membranes, and bodies of duck embryos dead within 24–48 hpi were harvested, homogenized and clarified. The titer of the virus was determined to be 10^5.7^ 50% egg lethal doses (ELD_50_) per 0.2 ml.

### Animals

The G1 generations of the resistant Pekin duck stock (Z8R1) and of the susceptible Pekin duck stock (Z8S1) were created by Zhang et al. (2016). The ducks were kept in Peking duck breeding farm, Institute of Animal Sciences, Chinese Academy of Agricultural Sciences, Beijing, China.

### Family Creation and Management

When the G1 ducks grew to approximately 24 weeks of age, 8 males and 32 females with good performance were collected from the Z8R1 flock to construct 32 families. The females were fed separately, and each male mated only with four fixed females. When the ducks grew to 30 weeks of age, eggs were collected continuously for 14 days and marked according to pedigree information. Two hundred and sixty-five offspring were produced and designated the G2 generation of the resistant Pekin duck stock (Z8R2). For comparison, we built 11 susceptible families in the same way, by selection of 11 females and 3 males ducks from the Z8S1 stock. Subsequently, 115 offspring were hatched and designated G2 generation of the susceptible Pekin duck stock (Z8S2). The ducks in the Z8R2 and Z8S2 groups were marked with wing-tag according to their pedigree information. Additionally, 50 1-day-old Pekin ducks were selected from a specialized strain Z7 and served as mock-infected control group in infection experiments. All ducklings were tested DHAV-3 antibody-free by indirect enzyme-linked immunosorbent assay (iELISA).

### Animal Experiments

Animal experiments were approved by the animal care and use committee of Institute of Animal Sciences of Chinese Academy of Agricultural Sciences. When ducklings grew to 7 days of age, groups Z8S2 and Z8R2 were inoculated intramuscularly with strain 112803 at the dose of 10^5.7^ ELD_50_ per birds. Group Z7 was injected intramuscularly with 0.2 ml of sterile phosphate-buffered saline (PBS). The ducklings were kept in isolation room and monitored every day. Dead ducklings were immediately examined for lesions.

### Histopathological Examination

Liver tissues were collected from ducks in groups Z8S2 and Z8R2 at 30 hpi and fixed in 4% buffered formaldehyde solution (pH 7.4) for 24 h. For each group, two live ducks and two dead ducks were employed. Following dehydration by using different concentrations of alcohol, the tissues were embedded in paraffin, and cut into 5-μm-thick sections. The sections were stained with hematoxylin-eosin (H.E.) and observed under light microscopy (Olympus, Japan).

### Serum Biochemistry Analysis

Live ducks were randomly selected from groups Z8R2 and Z7 at 1, 6, 12, 24, 36, 48, and 60 hpi, and from group Z8S2 at 1, 6, and 12 hpi, respectively. At each sampling time, five birds were selected from each group for serum collection. In group Z8S2 three ducklings exhibited clinical signs typical of DVH at 22 hpi. Serum samples were collected from the three diseased ducks. Since 24 hpi, high mortality occurred in group Z8S2, and we stopped collecting serum samples from this group.

The serum samples were analyzed for biochemical markers, including aspartate aminotransferase (AST), alanine aminotransferase (ALT), alkaline phosphatase (ALP), and γ-glutamyltransferase (GGT) (Hyder et al., 2013) using diagnostics reagents (Maccura, Sichuan, China) according to the manufacturer’s instructions. The analysis was performed using auto-analyzer (Hitachi 7080 Automate, Tokyo, Japan).

### Detection of Viral Load

Ducks used for serum collection were euthanatized, and their livers were sampled. Livers were also collected from two ducks died at 24 hpi and 15 ducks died between 24 and 60 hpi in group Z8S2. When the mortalities of the groups Z8S2 and Z8R2 were available, liver samples collected from families of Z8S2 showing high mortality and from families of Z8R2 survived DHAV-3 infection were used for detection of virus load. In addition, organs (e.g., liver, heart, and spleen) were sampled at 30 hpi from five live ducks in group Z8R2 and five dead ducks in group Z8S2 for detection of virus load.

On the basis of the VP1 nucleotide sequence of the 112803 isolate of DHAV-3, primers (**Table 1**) were designed and used for development of SYBR Green real time PCR. RNA was extracted from the virus using an RNeasy Mini kit (Qiagen, Hilden, Germany) and eluted in 50 μl of RNase-Free water. Five microliter of RNA was mixed with 20 pmol of reverse primer, incubated at 70°C for 5 min, and then chilled on ice. Subsequently, 5 μl of 5× RT buffer (Promega, Madison, WI, United States), 1 μl of RNase inhibitor (40 U/μl; TaKaRa, Dalian, China), 1 μl of M-MLV reverse transcriptase (200 U/μl; Promega, Madison, WI, United States), 5 μl of each dNTP mix (10 mM; TianGen, Beijing, China), and ddH_2_O were added in a final volume of 25 μl. The reaction mixture was incubated at 42°C for 1 h, followed by incubation at 94°C for 5 min. Five microliter of the cDNA was mixed with 12.5 μl of Taq plus PCR MasterMix (TianGen, Beijing, China), 1 μl of each of forward and reverse primers (20 pmol), and 5.5 μl of ddH_2_O. PCR was performed using cycling conditions as follows: 5 min at 94°C, followed by 35 cycles of 30 s at 94°C, 30 s at 60°C, and 30 s at 72°C, and a final extension step for 10 min at 72°C. PCR products were purified using an EasyPure Quick Gel Extraction Kit (TransGen, Beijing, China), according to the manufacturer’s instructions. Purified PCR fragments were cloned into pGEM-T Easy Vector (Promega, Madison, WI, United States) and transformed into *Escherichia coli* DH5a competent cells (TransGen, Beijing, China). Following incubation, plasmid DNAs were extracted from the separated insert-positive clones and purified by EasyPure Plasmid MiniPrep Kit (TransGen, Beijing, China). The purified plasmid was linearized by Pst I (TaKaRa, Dalian, China) digestion, according to the manufacturer’s instructions.

**Table 1:** Primers used in real-time quantitative RT-PCR assay.

Target	Forward primer (5′–3′)	Reverse primer (5′–3′)	Accession no.
GAPDH	ATGTTCGTGATGGGTGTGA	CTGTCTTCGTGTGTGGCTGT	AY436595
TLR3	GCAACACTCCGCCTAAGTATCA	CAGTAGAAAGCTATCCTCCACCCT	NM_001310782
TLR4	TGGCAGGGCTACAGGTCAAC	GCTCTGGGTAATACGAAGCACTCT	NM_001310413
TLR7	GACAACCTTTCCCAGAGCATTC	ACAGCCTTTTCCTCAGCCTAAC	DQ888645
RIG-I	CAGGTATGACCCTCCCAAGC	CGGAGTATTCATAGAGCACAACAAG	KP981415
MDA5	CGAGGAGGCTGACCACGAC	TTCACGCAGAGCAACCAAGA	NM_001310811
IL-2	TTCCCTGAATTTCGCCAAGA	CCCAAAGCGGACAGCAAG	XM_005015555
IL-6	CCAAGGTGACGGAGGAAGAC	GTGAGGAGGGATTTCTGGGTAG	AB191038
IL-8	GGCATCGGTGTTCTTATCTTCG	CCACGGGCTGACTGTGACTAA	AB236334
IFN-α	AGAACCTGCCCAGTCCTACGG	GTGCCTGCTGTGCTGACGG	X84764
IFN-γ	GGTGATGTTTACCAAGTTTCCGT	GTTGCCAAGTAGCCTGTCCTCT	AF087134
D3VP1	CCGAGAAGGCACAAGAAGCAA	TTAGTCCACCAGCAGCACCAGAT	EU352805


The concentration of the linearized plasmid was measured by an ultraviolet spectrophotometer (NanoDrop, Thermo Scientific, Paisley, United Kingdom). For the external standard curve, serial dilutions (between 10^9.94^ to 10^2.23^ molecules) of the plasmid were used as templates. Three microliter of template was mixed with 10 μl of SYBR^®^ Premix Ex Taq^TM^ II (TaKaRa, Dalian, China), 10 mM of each forward and reverse primer, and ddH_2_O in a final volume of 20 μl. Quantitative real-time PCR (qPCR) was carried out at 94°C for 30 s, followed by 40 cycles of 94°C for 5 s and 60°C for 30 s, a cycle of 95°C for 10 s, and a final program of 60°C to 95°C for 350 s (The duration for every 0.5°C increment of temperature was 5 s).

For determination of viral load, 500 ng tissue-extracted RNA was treated with 1 μl of DNase I (TaKaRa, Dalian, China) to remove DNA. Then 2 μl of 5× PrimeScript RT Master Mix (TaKaRa, Dalian, China) and ddH_2_O were added in a final volume of 10 μl. For synthesis of cDNA, the reaction mixture was incubated at 37°C for 15 min, followed by incubation at 85°C for 5 s. qPCR was performed as described above.

### Detection of Expression of Immune-Associated Genes

Liver samples used for detection of viral load were also used for detection of expression of immune-associated genes. Since deaths in group Z8S2 occurred at 24 hpi, we only detected the samples collected between 1–24 hpi. The expression of immune-related genes, including toll-like receptor (TLR) 3, TLR4, TLR-7, retinoic acid-inducible gene-I (RIG-I), melanoma differentiation-associated gene 5 (MDA-5), interleukin (IL)-2, IL-6, IL-8, interferon (IFN)-α and IFN-γ, were evaluated by relative qRT-PCR with primers listed in **Table 1**. Following optimization of reaction conditions (e.g., primer and reagent concentrations, annealing temperatures and elongation intervals), 500 ng liver-extracted RNA was reverse transcribed and subjected to qRT-PCR as described above, except that annealing at 57–60°C was performed. To confirm the specificity of amplification, PCR product was sequenced after cloning as described above.

### Statistical Analysis

The relative expression levels of immune-related genes in livers collected from the Z8R2 and Z8S2 flocks were evaluated by the 2^-ΔΔCT^ method (Livak and Schmittgen, 2001), using glyceraldehyde-3-phosphate-dehydrogenase (GAPDH) as endogenous reference gene (Adams et al., 2009). The expression level of Z7 group was normalized to a level of 1 (baseline). Each liver sample was tested in triplicate. Biochemical marker value, the virus RNA copy and immune-related gene mRNA fold change value between Z8R2 and Z8S2 groups were compared by analysis of variance using Student’s *t*-test in the GraphPad Prism 5.0 program (GraphPad Prism Software, United States). All data were calculated as mean values ± standard deviation (SD). A significance level of *P <* 0.05 was employed.

## Results

### Response of Pekin Ducks to DHAV-3 Infection

**FIGURE 1:**
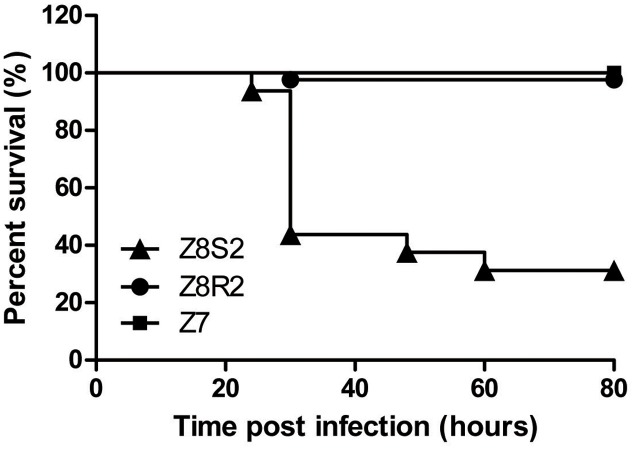
Survival curves of flocks Z8S2 and Z8R2 after inoculated with virulent strain of DHAV-3. Flock Z8S2 and Z8R2 ducklings were infected intramuscularly with 10^5.7^ ELD_50_ of DHAV-3 112803 strain. Flock Z7 ducklings were infected intramuscularly with 0.2 ml of sterile phosphate-buffered saline (PBS). Mortality occurred between 24 and 60 h after infection in Z8S2 flocks and at 30 hpi in Z8R2 flocks.

To confirm the presence of difference in response to DHAV-3 between Pekin duck Z8S2 and Z8R2 flocks, we conducted infection experiments with strain 112803 at the dose of 10^5.7^ ELD_50_ per birds. High mortality (66.3%) was observed in flock Z8S2, in sharp contrast with the extremely low mortality rate (2.67%) in flock Z8R2. Mortality in flock Z8S2 occurred at 24–60 hpi, with most deaths (49/98) on the second day (24–30 hpi) (**Figure 1**). Of 11 families, death appeared in 10 families, and losses of 62.5–90.9% occurred in nine families (**Table 2**). In flock Z8R2 mortality occurred at 30 hpi (**Figure 1**). Of 32 families, death appeared in only two families, with mortalities of 36.4% (4/11) and 40% (2/5) respectively (**Table 2**).

**Table 2:** Mortality occurred in families of Z8S2 and Z8R2 groups of ducklings infected with DHAV-3.

Group	No. of families	No. of individuals^a^	No. of death	Mortality (%)^b^
Z8S2	556	10	9	90
	638	5	4	80
	639	7	6	85.7
	640	11	8	72.7
	642	8	7	87.5
	643	9	8	88.9
	644	10	1	10
	646	11	10	90.9
	649	10	7	70
	651	9	0	0
	652	8	5	62.5

Z8R2	547	5	0	0
	548	10	0	0
	549	9	0	0
	550	11	0	0
	551	7	0	0
	553	11	4	36.4
	555	7	0	0
	557	5	0	0
	559	9	0	0
	560	7	0	0
	561	8	0	0
	562	8	0	0
	564	5	0	0
	565	10	0	0
	566	7	0	0
	567	4	0	0
	568	5	0	0
	569	8	0	0
	570	4	0	0
	571	9	0	0
	573	5	0	0
	574	7	0	0
	577	5	0	0
	579	8	0	0
	581	10	0	0
	582	4	0	0
	584	5	0	0
	587	7	0	0
	590	4	0	0
	591	8	0	0
	593	8	0	0
	594	5	2	40


*^a^Excluding ducklings used for sample collection. ^b^No. of death/no. of individuals inoculated.*

In flock Z8S2 most of the infected ducklings exhibited clinical signs typical of DVH, including lethargy, ataxia, and sudden death with opisthotonos (Woolcock and Fabricant, 1991). While most of the infected ducklings in flock Z8R2 didn’t show obvious clinical signs. Only six ducklings exhibited clinical signs typical of DVH.

**FIGURE 2:**
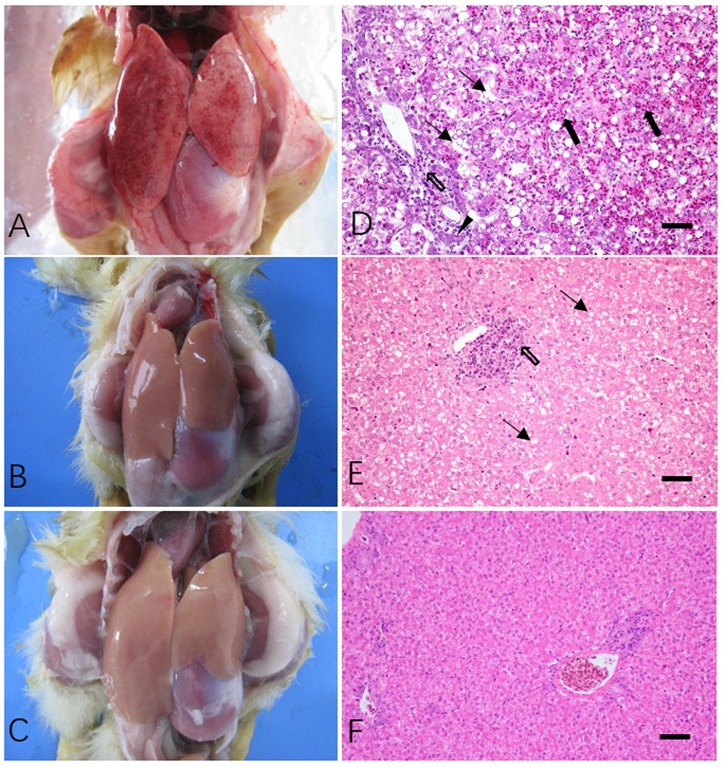
Gross and microscopic lesions of livers in Z8S2 and Z8R2 ducklings infected with DHAV-3 at 30 hpi. **(A)** Redness and numerous hemorrhagic spots in livers from dead ducklings of Z8S2 and Z8R2 flocks. **(B)** No significant cross lesions in livers from live ducklings of Z8S2 and Z8R2 flocks. **(C)** A liver from mock-infected ducklings. **(D)** Hepatocyte necrosis (arrow), hemorrhage, congestion (black arrow), bile duct hyperplasia (triangle) and significant inflammatory cell infiltration (hollow arrow) in dead ducklings of both Z8S2 and Z8R2 flocks. **(E)** Slight vacuolar degeneration (arrow) and focal lymphocytic aggregations (hollow arrow) in live ducklings of both Z8S2 and Z8R2 flocks. **(F)** No gross and microscopic lesions in livers of mock-infected ducklings. Bar = 50 μm.

At necropsy multiple hemorrhages were found in the liver of dead cases in both Z8S2 and Z8R2 flocks (**Figure 2A**). No cross lesions were seen in live ducklings in both infected flocks Z8R2 and flock Z7 (**Figures 2B,C**), Histopathologically, the liver collected from all dead ducklings in Z8S2 and Z8R2 flocks exhibited lesions typical of DVH, including extensive hepatocyte necrosis, bile duct hyperplasia, hemorrhage, congestion and significant inflammatory cell infiltration (**Figure 2D**). In live ducklings in flock Z8R2, only slight vacuolar degeneration and focal lymphocytic aggregations could be observed in the livers (**Figure 2E**). No gross and microscopic lesions were observed in mock-infected control (**Figure 2F**).

### Detection of Biochemical Markers in Serum Samples

**FIGURE 3:**
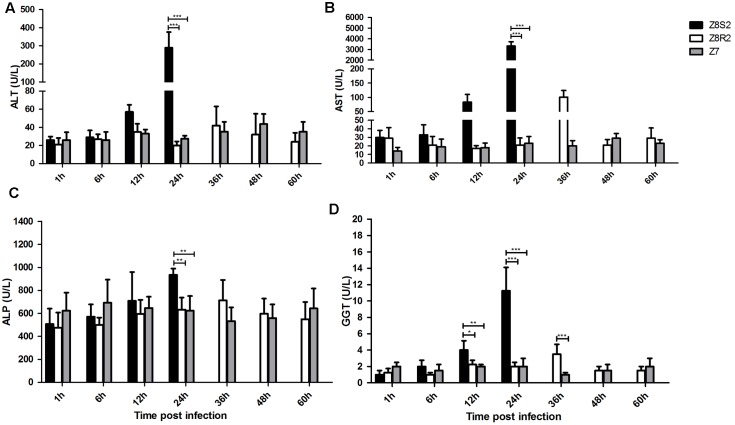
Detection of serum biochemical markers ALT **(A)**, AST **(B)**, ALP **(C)** and GGT **(D)**. For group Z8S2, sera were sampled from live ducklings between 1 and 12 h and from ducklings showing clinical signs at 22 h. For groups Z8R2 and Z7, sera were sampled from live ducklings between 1 and 60 h. Groups Z8S2, Z8R2 and Z7 were compared with each other for statistical significant differences using two-way ANOVA followed by Student’s *t*-test. ^∗^*P* < 0.05; ^∗∗^*P* < 0.01; ^∗∗∗^*P* < 0.001.

Serum biochemical markers (ALT, AST, ALP and GGT) were detected to evaluate the degree of hepatic injury in Z8S2 and Z8R2 groups following infection with DHAV-3. As shown in **Figure 3**, for the four markers, high levels were detected at 24 hpi in Z8S2, which were significantly higher than those of Z8R2 and Z7 (*P* < 0.01). For GGT, the level in Z8S2 was significantly higher than those of Z8R2 and Z7 at 12 hpi (*P* < 0.05), and the level of GGT in Z8R2 was significantly higher than those of Z7 groups at 36 hpi (*P* < 0.001). There was no significant difference among groups when the biochemical markers in remaining samples were analyzed (*P* > 0.05).

### Viral Load Analysis

**FIGURE 4:**
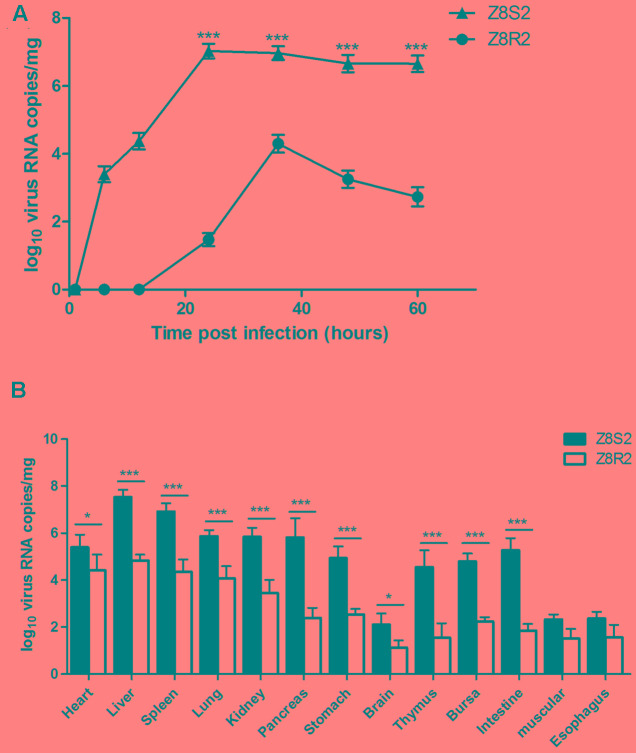
Viral load in Z8S2 and Z8R2 groups of ducklings infected with DHAV-3. **(A)** RNA copy number in livers of ducklings between 1 and 60 h after infection. For group Z8S2, 3 live ducklings were used for 1 and 6 h, 5 for 12 h, and 5 dead ducklings for each sampling time between 24 and 60 h. For sample collection, ducklings dead between 22 and 24 h, 24 and 36 h, 36 and 48 h, and 48 and 60 h were recorded as those died at 24, 36, 48, and 60 h, respectively. For flock Z8R2, 5 live ducklings were used for each sampling time except 4 for 6 and 48 h. **(B)** RNA copy number in different organs of ducklings at 30 hpi. Five dead ducklings were used in group Z8S2, and 5 live ducklings were used in group Z8R2. Groups Z8S2 and Z8R2 were compared for statistical significant differences using two-way ANOVA followed by Student’s *t*-test. ^∗^*P* < 0.05; ^∗∗∗^*P* < 0.001.

To determine whether the host background could influence on DHAV-3 replication, a DHAV-3 VP1-based qRT-PCR was employed to investigate differences in the kinetics of the virus loads in livers between resistance and susceptible ducklings. As shown in **Figure 4A**, DHAV-3 was detected in the liver of ducklings in flock Z8S2 as early as 6 hpi (10^3.36^ copies). Then the virus load increased at 12 hpi (10^4.37^ copies), and peaked at 24 hpi (10^7.37^ copies). High levels of virus load persisted to 60 hpi (10^6.65^ copies) with a slight decline. By contrast, the virus could not be detected in the liver of ducklings in flock Z8R2 until 24 hpi, with a low level of virus load (10^1.47^ copies). The highest concentration of the virus (10^4.29^ copies) was observed at 36 hpi. Then the virus load dropped rapidly, and fell to 10^2.73^ copies at 60 hpi. In sampling time of 24 to 60 hpi, the viral load detected from flock Z8S2 was significantly higher than those from flock Z8R2 (*P* < 0.001).

We also investigated virus load in different tissues in groups Z8S2 and Z8R2 at 30 hpi, when the deaths reached a peak in group Z8S2 (**Figure 4B**). DHAV-3 was detected from all tissues collected from ducklings in both Z8S2 and Z8R2 groups. However, apart from muscular and esophagus, the virus loads in most of the tissues in group Z8S2 were significantly higher than those in group Z8R2 (*P* < 0.05). In both Z8S2 and Z8R2 groups the highest levels of virus load (10^7.52^ and 10^4.83^ copies, respectively) were detected in the liver. The levels of virus RNA detected in most of the tissues (except brain, muscular and esophagus) in group Z8S2 were generally high, ranging from 10^4.55^ to 10^6.92^ copies. In group Z8R2, however, relatively higher levels of virus load (10^3.46^ to 10^4.83^ copies) were detected only from liver, heart, spleen, lung, and kidney when compared with those from all other tissues, which harbored low levels of virus load (10^1.12^ to 10^2.53^ copies).

### Analysis on Expression of PRRs Genes

To understand difference in the innate immune response to DHAV-3 infection between Z8S2 and Z8R2 flocks, we detected the expression of PRRs, including TLR 3/4/7, RIG-I and MDA5 in the liver.

**FIGURE 5:**
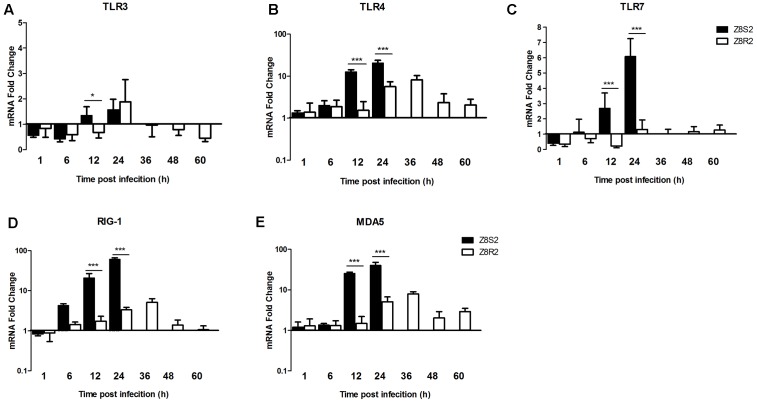
Expression of PRRs in livers of Z8S2 and Z8R2 groups of ducklings infected with DHAV-3. Fold changes of TLR3 **(A)**, TLR4 **(B)**, TLR7 **(C)**, RIG-I **(D)**, and MDA5 **(E)** were calculated using the 2^-ΔΔCT^ method. Groups Z8S2 and Z8R2 were compared for statistical significant differences using two-way ANOVA followed by Student’s *t*-test. For group Z7, the samples used for detection of serum collection were employed. For groups Z8S2 and Z8R2, the samples used for detection of viral load were employed except those collected between 36 and 60 h after infection in group Z8S2. ^∗^*P* < 0.05; ^∗∗∗^*P* < 0.001.

The TLR3 expression was down-regulated in the early stage of infection (1 and 6 hpi) and up-regulated at 24 hpi (1.58- and 1.88-fold, respectively) in both Z8S2 and Z8R2 groups when compared with group Z7 (**Figure 5A**). At 1, 6, and 24 hpi, there was no significant difference between Z8S2 and Z8R2 groups (*P* > 0.05). In the Z8R2 group down-regulation of the TLR3 expression was found between 36 to 60 hpi. Significant difference (*P* < 0.05) between Z8S2 and Z8R2 groups was seen at 12 hpi, when the expression of TLR3 was up-regulated in group Z8S2 (1.35-fold), and down-regulated in group Z8R2 (0.68-fold). Nevertheless, the levels of up- or down-regulation of the TLR3 expression in both groups were very low.

In group Z8S2 expression of TLR4/7, RIG-I and MDA5 changed similarly, all of which increased remarkably at 12 hpi (12.68-, 2.70-, 20.91- and 25.69-fold, respectively), and peaked at 24 hpi (20.46-, 6.09-, 60.99- and 40.48-fold, respectively) (**Figures 5B–E**). In the Z8R2 group expression of TLR4, RIG-I and MDA5 also changed similarly. They were moderately up-regulated at 24 hpi (5.63-, 3.34-, and 5.06-fold, respectively), peaked at 36 hpi (8.18-, 5.12-, and 7.92-fold, respectively), and decreased at 48–60 hpi (**Figures 5B,D,E**). The levels of the TLR4, RIG-I and MDA5 expression in group Z8R2 at 24 and 36 hpi were significantly lower than those in group Z8S2 at 12 and 24 hpi (*P* < 0.001).

Compared to group Z7, the expression of TLR7 in group Z8R2 was down-regulated between 1 and 12 h after infection, and up-regulated between 24 and 60 h after infection. Nevertheless, the levels of the TLR7 expression in group Z8R2 were close to the baseline from 1 to 60 hpi (**Figure 5C**).

### Detection of Cytokine Expression

To investigate the immunological basis associated with susceptibility and resistance of Pekin ducks, we detected difference in expression levels of pro-inflammatory cytokines (IL-2, IL-6), chemokine (IL-8) and IFN type I and II (IFN-α, IFN-γ) in the liver of ducklings between Z8S2 and Z8R2 flocks following DHAV-3 infection.

**FIGURE 6:**
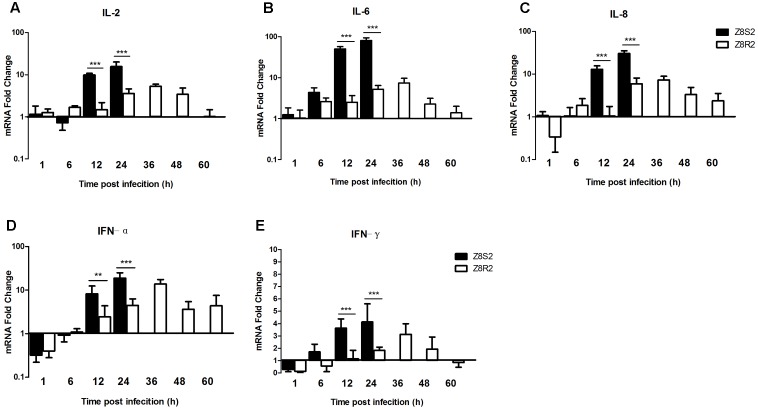
Expression of cytokines in livers of Z8S2 and Z8R2 groups of ducklings infected with DHAV-3. Fold changes of IL-2 **(A)**, IL-6 **(B)**, IL-8 **(C)**, IFN-α **(D)**, and IFN-γ **(E)** were calculated using the 2^-ΔΔCT^ method. Groups Z8S2 and Z8R2 were compared for statistical significant differences using two-way ANOVA followed by Student’s *t*-test. For group Z7, the samples used for detection of serum collection were employed. For groups Z8S2 and Z8R2, the samples used for detection of viral load were employed except those collected between 36 and 60 h after infection in group Z8S2. ^∗∗^*P* < 0.01; ^∗∗∗^*P* < 0.001.

In flock Z8S2 the expression levels of IL-2, IL-8 and IFN-α displayed a similar up-regulation pattern, with significant increase by 9.84- and 14.79, 12.98- and 30.45, and 8.42- and 18.94 at 12 and 24 hpi, respectively (**Figures 6A,C,D**). While the up-regulation of IL-6 and IFN-γ expression appeared at 6 hpi (4.39- and 1.72-fold), increased greatly at 12 hpi (50.63- and 3.63-fold), and peaked at 24 hpi (80.85- and 4.14-fold) (**Figures 6B,E**).

In group Z8R2 the expression patterns of IL-2, IL-6, IL-8, IFN-α, and IFN-γ were similar to one another, in which up-regulation occurred at 24 hpi (3.57-, 5.22-, 5.92-,4.47-, and 1.83-fold, respectively), reached a peak at 36 hpi (5.31-, 7.44-, 7.33-, 13.86-, and 3.13-fold, respectively), and declined from 48 to 60 hpi (**Figures 6A–E**). The expression levels of all cytokines in the Z8S2 group at 12 and 24 hpi, were significantly higher than those in the Z8R2 group detected at the same time (*P* < 0.01).

## Discussion

The present paper reports the host response of the second generation of the Pekin ducks Z8 line to DHAV-3 infection. We showed that infection at 7 days resulted in high mortality in the Z8S2 flock and an extremely low mortality in the Z8R2 flock, indicating that flock Z8S2 is highly susceptible to DHAV-3, while flock Z8R2 displays strong resistance to the virus. The possibility that survival of most ducklings in flock Z8R2 after infection may be attributed to maternal immunity can be ruled out based on the following points. First, for selection of the Z8R1, the original duck flock was infected with DHAV-3 at 7 days of age. The antibody induced by the infection cannot be persisted to 24 weeks of age. Second, all tested ducks were not immunized with vaccine. Third, the half-time of maternal antibodies in the ducklings transferred from parent flocks is 4 days (Wu and Guo, 1994). Finally, using a DHAV-3-specific ELISA, the serum antibodies in flocks Z8R2, Z8S2 and Z7 were tested negative. Together these findings demonstrate clearly that the resistance of Z8R1 to DHAV-3 has been transmitted to Z8R2. We also noted that six ducklings in Z8R2 died from infection, and that the dead ducklings exhibited lesions typical of duck hepatitis. This suggests that, of the 32 families of Z8R2, a few individuals in two families show a weak resistance to DHAV-3. It is therefore necessary to produce more generations of the resistant Pekin ducks employing the method of family selection together with infection experiments, which may be of help to construction of a stable resistant line.

The causative agents of DVH include three genotypes of DHAV and two different duck astroviruses. Among these viruses, DHAV-3 is the virus strain reported in 2007 (Kim et al., 2007). To date, little is known about the pathogenesis of the virus. Like other four viruses, the principle lesions caused by DHAV-3 are found in the liver, which contains punctate or ecchymotic hemorrhages, suggesting that the main target organ may be liver. Detection of DHAV-3 in multiple organs of infected ducklings in both Z8S2 and Z8R2 groups suggests that the virus may have a wide range of tissue tropism. Significantly more viral RNA copies were detected in the liver than in other organs of ducklings collected at 30 hpi, supported the view that the main organ target is liver. Comparative analysis revealed that the virus RNA in the liver was detected earlier from Pekin duck Z8S2 (6 hpi) than from Pekin duck Z8R2 (24 hpi). Moreover, in each sampling time the viral loads in the liver detected from Z8S2 were significantly higher than those of Z8R2. Together these findings suggest that the DHAV-3 strain can be replicated rapidly and efficiently in the liver of Pekin duck Z8S2, whereas the replication of the virus in the liver of Pekin duck Z8R2 is suppressed greatly. This implies that the resistance and susceptible of Pekin ducks to DHAV-3 is closely related to the reproductive efficiency of the virus in the liver.

Recognition of viruses by PRRs is crucial for initiation of host innate immune response. So far seven PRRs have been identified in ducks (Brennan and Bovie, 2010; Chen et al., 2013). In the present study, we showed that TLR4, RIG-I and MDA5 were expressed in the liver in both Z8S2 and Z8R2 Pekin ducklings after infection, indicating the ducklings were able to mount a normal, early immune defense. At 12 and 24 hpi, however, the expression levels of TLR4/7, MDA5 and RIG-I in Z8S2 were significantly higher than those in Z8R2, indicating that susceptible Z8S2 Pekin ducks exhibit a much stronger innate immune response than resistant Z8R2 Pekin ducks. The analysis of transcripts in the liver revealed that the expression levels of inflammatory cytokines (e.g., IL-2, IL-6) and chemokines (e.g., IL-8) in Z8S2 were significantly higher than those in Z8R2, suggesting that susceptible Z8S2 Pekin ducks exhibits a much stronger inflammatory response than resistant Z8R2. Previous works have shown that highly pathogenic avian influenza viruses cause strongly elevated levels of cytokines and chemokines in mammals and birds, resulting in detrimental immune pathologies and tissue damage (Srivastava et al., 2009; Suzuki et al., 2009; Wei et al., 2013; Burggraaf et al., 2014; Oldstone and Rosen, 2014). It is likely that the severe liver pathology and high mortality in Z8S2 ducks may be correlated with the strong innate immune response and inflammatory response. In general, IFN-α and IFN-γ play key roles in anti-virus response (Takeuchi and Akira, 2010). However, overexpression of IFN-γ in the liver in ducks infected by DHAV-1 may have some relationship with liver damage and apoptosis of hepatic cells (Song et al., 2014). We showed that the expression levels of IFN-α and IFN-γ in Z8R2 were significantly lower than those in Z8S2 (*P* < 0.01), suggesting that the expression of IFN-α and IFN-γ is related to susceptibility of Pekin ducks to DHAV-3.

## Conclusion

Z8R2 and Z8S2 Pekin ducklings, which were derived from the same Z8 line, exhibit disparate pathogenic outcomes following DHAV-3 infection. Therefore, it is possible to select a Pekin duck flock resistant to DHAV-3 employing the strategies described here. It is likely that the high viral load and the strong inflammatory response correlate with the high susceptibility of the Z8S2 Pekin ducks to DHAV-3. Further studies, including identification of critical genomic regions responsible for susceptibility and/or resistance to DHAV-3, are needed to better understand the resistance mechanism of Z8R2 and pathogenic mechanism of Z8S2 associated with DHAV-3 infections.

## Author Contributions

SH and XW conceived and designed the study. JZ and XW performed experiments. RM, SL, YJ, and YZ helped with the animal experiments. MX, ZZ, and XW analyzed data. SH and XW wrote the paper.

## Conflict of Interest Statement

The authors declare that the research was conducted in the absence of any commercial or financial relationships that could be construed as a potential conflict of interest.

## Footnotes

1 http://www.picornaviridae.com/avihepatovirus/avihepatovirus_a/avihepatovirus_a.htm

## References

[B1] Adams S. C. Xing Z. Li J. Cardona C. J. (2009). Immune-related gene expression in response to H11N9 low pathogenic avian influenza virus infection in chicken and Pekin duck peripheral blood mononuclear cells. *Mol. Immunol.* 46 1744–1749. 10.1016/j.molimm.2009.01.025 19250679

[B2] Asplin F. D. (1965). Duck hepatitis: vaccination against two serological types. *Vet. Rec.* 77 1529–1530. 10.1136/vr.77.50.1529 5849750

[B3] Bosch A. Guix S. Krishna N. K. Méndez E. Monroe S. S. Pantin-Jackwood M. (2011). “Family astroviridae,” in *Virus Taxonomy. Classification and Nomenclature of Viruses. Ninth Report of the International Committee on Taxonomy of Viruses*, eds King A. M. Q. Adams M. J. Carstens E. B. Lefkowitz E. J. (London: Elsevier), 953–959.

[B4] Brennan K. Bovie A. G. (2010). Activation of host pattern recognition receptors by viruses. *Curr. Opin. Microbiol.* 13 503–507. 10.1016/j.mib.2010.05.007 20538506

[B5] Bumstead N. Barrow P. (1993). Resistance to *Salmonella gallinarum*, *S. pullorum*, and *S. enteritidis* in inbred lines of chickens. *Avian Dis.* 37 189–193. 10.2307/1591473 8452495

[B6] Bumstead N. Reece R. L. Cook J. K. (1993). Genetic differences in susceptibility of chicken lines to infection with infectious Bursal Disease Virus. *Poult. Sci.* 72 403–410. 10.3382/ps.0720403 8385328

[B7] Burggraaf S. Karpala A. J. Bingham J. Lowther S. Selleck P. Kimpton W. (2014). H5N1 infection causes rapid mortality and high cytokine levels in chickens compared to ducks. *Virus Res.* 185 23–31. 10.1016/j.virusres.2014.03.012 24657784 PMC7127704

[B8] Chen S. Cheng A. Wang M. (2013). Innate sensing of viruses by pattern recognition receptors in birds. *Vet. Res.* 44:82 10.1186/1297-9716-44-82 PMC3848724 24016341

[B9] Doan H. T. Le X. T. Do R. T. Hoang C. T. Nguyen K. T. Le T. H. (2016). Molecular genotyping of duck hepatitis A viruses (DHAV) in Vietnam. *J. Infect. Dev. Ctries.* 10 988–995. 10.3855/jidc.7239 27694732

[B10] Fu Y. Pan M. Wang X. Xu Y. Yang H. Zhang D. (2008). Molecular detection and typing of duck hepatitis A virus directly from clinical specimens. *Vet. Microbiol.* 131 247–257. 10.1016/j.vetmic.2008.03.011 18462894

[B11] Hyder M. A. Hasan M. Mohieldein A. H. (2013). Comparative levels of ALT, AST, ALP and GGT in liver associated diseases. *Eur. J. Exp. Biol.* 3 280–284. 10.9790/0853-0757275

[B12] Kim M. C. Kim M. J. Kwon Y. K. Lindberg A. M. Joh S. J. Kwon H. M. (2009). Development of duck hepatitis A virus type 3 vaccine and its use to protect ducklings against infections. *Vaccine* 27 6688–6694. 10.1016/j.vaccine.2009.08.092 19747575

[B13] Kim M. C. Kwon Y. K. Joh S. J. Kim S. J. Tolf C. Kim J. H. (2007). Recent Korean isolates of duck hepatitis virus reveal the presence of a new geno- and serotype when compared to duck hepatitis virus type 1 type strain. *Arch. Virol.* 152 2059–2072. 10.1007/s00705-007-1023-0 17701025

[B14] Knowles N. J. Hovi T. Hyypiä T. King A. M. Q. Lindberg A. M. Pallansch M. A. (2012). “Family picornaviridae,” in *Virus Taxonomy. Classification and Nomenclature of Viruses. Ninth Report of the International Committee on Taxonomy of Viruses*, eds King A. M. Q. Adams M. J. Carstens E. B. Lefkowitz E. J. (London: Elsevier), 855–880.

[B15] Lee L. F. Powell P. C. Rennie M. Ross L. J. Payne L. N. (1981). Nature of genetic resistance to Marek’s disease in chickens. *J. Natl. Cancer Inst.* 66 789–796. 10.1093/jnci/66.4.789 6262555

[B16] Lin S. L. Cong R. C. Zhang R. H. Chen J. H. Xia L. L. Xie Z. J. (2016). Circulation and in vivo distribution of duck hepatitis A virus types 1 and 3 in infected ducklings. *Arch. Virol.* 161 405–416. 10.1007/s00705-015-2648-z 26597185

[B17] Livak K. J. Schmittgen T. D. (2001). Analysis of relative gene expression data using real-time quantitative PCR and the 2^-ΔΔ^*^C^*_T_ method. *Methods* 25 402–408. 10.1006/meth.2001.1262 11846609

[B18] Oldstone M. B. Rosen H. (2014). Cytokine storm plays a direct role in the morbidity and mortality from influenza virus infection and is chemically treatable with a single sphingosine-1-phosphate agonist molecule. *Curr. Top. Microbiol. Immunol.* 378 129–147. 10.1007/978-3-319-05879-5_6 24728596 PMC7121493

[B19] Soliman M. Aifajaro M. M. Lee M. H. Jeong Y. J. Kim D. S. Son K. Y. (2015). The prevalence of duck hepatitis A virus types 1 and 3 on Korean duck farms. *Arch. Virol.* 160 493–498. 10.1007/s00705-014-2264-3 25359107

[B20] Song C. Liao Y. Gao W. Yu S. Sun Y. Qiu X. (2014). Virulent and attenuated strains of duck hepatitis A virus elicit discordant innate immune responses in vivo. *J. Gen. Virol.* 95 2716–2726. 10.1099/vir.0.070011-0 25217614

[B21] Srivastava B. Błazejewska P. Hessmann M. Bruder D. Geffers R. Mauel S. (2009). Host genetic background strongly influences the response to influenza A virus infections. *PLoS ONE* 4:e4857 10.1371/journal.pone.0004857 PMC2654507 19293935

[B22] Suzuki K. Okada H. Itoh T. Tada T. Mase M. Nakamura K. (2009). Association of increased pathogenicity of Asian H5N1 highly pathogenic avian influenza viruses in chickens with highly efficient viral replication accompanied by early destruction of innate immune responses. *J. Virol.* 83 7475–7486. 10.1128/JVI.01434-08 19457987 PMC2708648

[B23] Takeuchi O. Akira S. (2010). Pattern recognition receptors and inflammation. *Cell* 140 805–820. 10.1016/j.cell.2010.01.022 20303872

[B24] Wei L. Jiao P. Song Y. Cao L. Yuan R. Gong L. (2013). Host immune responses of ducks infected with H5N1 highly pathogenic avian influenza viruses of different pathogenicities. *Vet. Microbiol.* 166 386–393. 10.1016/j.vetmic.2013.06.019 23920409

[B25] Woolcock P. R. (2003). “Duck hepatitis,” in *Diseases of Poultry*, 11th Edn, eds Saif Y. M. Barnes H. J. Glisson J. R. Fadly A. M. McDougald L. R. Swayne D. E. (Ames, IA: Iowa State Press), 343–354.

[B26] Woolcock P. R. Fabricant J. (1991). “Duck virus hepatitis,” in *Diseases of Poultry*, 9th Edn, eds Calnek B. W. Barnes H. J. Beard C. W. Reed W. M. Yoder H. W. (Ames, IA: Iowa State University Press), 597–608.

[B27] Wu T. Guo Y. (1994). Studies on ELISA for duck viral hepatitis. *Chin. J. Vet. Med.* 20 5–7.

[B28] Zhang D. Liu N. (2016). “7: Duck hepatitis virus,” in *Molecular Detection of Animal Viral Pathogens*, ed. Liu D. (Boca Raton, FL: CRC press), 53–60.

[B29] Zhang J. Jiang Y. Wang X. Zhou Z. Xie M. Hou S. (2016). The preliminary study of breeding for peking ducks resistance to duck hepatitis A virus type 3. *Chin. J. Anim. Sci.* 52 5–8.

